# Viral Versus Vaccine-Associated Acute Transverse Myelitis With Neuromyelitis Optica Immunoglobulin G Antibody and Myelin Basic Protein: A Case Report

**DOI:** 10.7759/cureus.28922

**Published:** 2022-09-08

**Authors:** Divya Mamootil, Anmol Grewal

**Affiliations:** 1 Internal Medicine, Ascension St. Agnes Hospital, Baltimore, USA; 2 Medicine, Ross University School of Medicine, Bridgetown, BRB

**Keywords:** coxsackie virus type b4, vaccine-associated transverse myelitis, echovirus type 6, multiple sclerosis, myelin basic protein, neuromyelitis optica, vaccine adverse effects, acute transverse myelitis

## Abstract

Transverse myelitis is a rare spinal cord disorder caused by local inflammation. Usually, this occurs as a complication from infection or autoimmune disease; however, there have been reported idiopathic causes such as vaccinations. A 73-year-old female with a medical history significant for Hashimoto’s thyroiditis presented with new-onset paresthesias in her lower extremities. Her symptom onset was about five weeks after receiving influenza and tetanus, diphtheria, and pertussis (TDaP) vaccines. Magnetic resonance imaging (MRI) of the spine revealed an increased T2 signal of the lower cervical and thoracic spine. Lumbar puncture was also performed, and cerebrospinal fluid (CSF) serology showed elevated myelin basic protein (MBP) at 108.3 ng/mL (reference range: 0-5.5 ng/mL). Serology panel revealed Coxsackie virus type B4 antibody at 1:80 (reference range: <1:10) and Echovirus type 6 antibody at 1:640 (reference range: <1:10). Neuromyelitis optica (NMO) immunoglobulin G (IgG) antibody was 24.6 U/mL (reference range: <2.9 U/mL). She was diagnosed with acute transverse myelitis (ATM) and treated with alternating steroids and plasma exchange (PLEX) therapy for five days each. This case highlights the possible associations of vaccines with transverse myelitis. Although ATM is a rare disorder with serious complications, it has a favorable prognosis in the setting of rapid detection and treatment. Vaccine-related ATM remains controversial, but patients with these adverse reactions need to be cautioned regarding potential recurrence risk.

## Introduction

Acute transverse myelitis (ATM) is a rare, non-compressive spinal cord disorder caused by local inflammation that can lead to a rapid onset of motor, sensory, and autonomic dysfunction [1]. Infectious and autoimmune etiologies are common causes of transverse myelitis; however, it can also be idiopathic [2]. Vaccine-related adverse effects are rare but have been reported over the years. Transverse myelitis can be associated with other neurological disorders such as multiple sclerosis (MS) or neuromyelitis optica (NMO) as well.

This case report discusses a patient with a previously diagnosed autoimmune disease who developed ATM about one month after receiving influenza and tetanus, diphtheria, and pertussis (TDaP) vaccinations. Prompt diagnosis and treatment of ATM are essential in preventing long-term neurological damage. It is important to take vaccination history into consideration when identifying patients with neurological complications to raise awareness of potentially dangerous adverse effects and guide future vaccination recommendations for these patients.

This article was presented as a clinical vignette poster at the American College of Physicians (ACP) Mulholland Mohler Resident Meeting on May 5, 2022.

## Case presentation

A 73-year-old Caucasian female with a medical history significant for Hashimoto’s thyroiditis, transient ischemic attack, and mitral valve prolapse presented to the emergency department with a complaint of paresthesias in her bilateral toes and difficulty ambulating. Her symptoms began as a burning sensation in her back along the T8 dermatome, prompting her to go to an urgent care center a few days later. There was a concern for shingles flare-up due to a history of shingles in her past, and she was started on valacyclovir with no improvement. She complained of constipation but denied fever, chills, urinary or bowel incontinence, vision changes, myalgias, arthralgias, or rashes. Her symptoms continued to worsen, with increased severity of pain even to light touch in her lower extremities up to the knees. Further history revealed she had been hiking a few weeks prior at Shenandoah Valley Park in Virginia and was unsure if she had any tick bites. She was vaccinated for COVID-19 and received her booster shot four days prior to admission. She had also recently received influenza and TDaP vaccines about a month prior to her symptom onset.

Upon arrival to the emergency room, she was afebrile with a pulse of 114 beats/minute, respiratory rate of 20 breaths/minute, blood pressure of 130/93 mmHg, and pulse oximetry of 100% on room air. On the physical examination, she was alert and oriented, cranial nerves II-XII were intact, and there was no nuchal rigidity. The strength on her right lower extremity was 3/5 throughout, while the left lower extremity was 5/5. At the T8 hemisensory level, there was decreased sensation to proprioception, vibration, and light touch in the left upper and lower extremity. Her reflexes were 3+ in bilateral lower extremities and 2+ in bilateral upper extremities. Plantar reflexes were extensor bilaterally. Her gait was unsteady and hemiparetic.

Laboratory findings on admission revealed blood glucose of 121 mg/dL (reference range: 65-105 mg/dL), white blood cell (WBC) of 11.5 K/uL (reference range: 4-11 K/uL) with 81% neutrophils and 1% eosinophils, hemoglobin of 16.5 g/dL (reference range: 12-15 g/dL), erythrocyte sedimentation rate (ESR) of 23 mm/hour (reference range: 0-20 mm/hour), C-reactive protein (CRP) of 8.4 mg/L (reference range: 0-5 mg/L), lactic acid of 2.2 mmol/L (reference range: 0.5-1.6 mmol/L), thyroid-stimulating hormone (TSH) of 11.11 mIU/L (reference range: 0.27-4.2 mIU/L), and free thyroxine (T4) of 1.98 ng/dL (reference range: 0.93-1.7 ng/dL).

**Figure 1:**
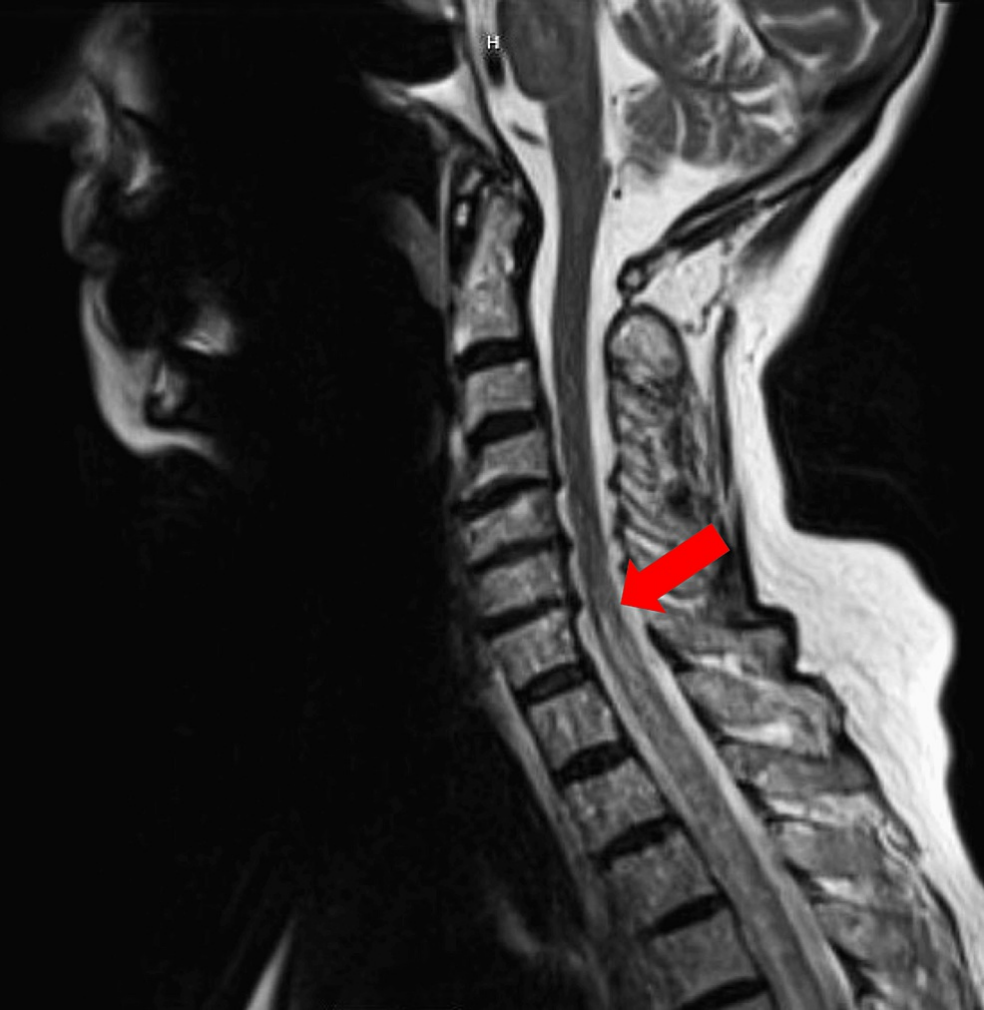
T2-weighted cervical spine MRI, sagittal view, showing increased T2 signal starting at C7 (arrow) MRI: magnetic resonance imaging

**Figure 2:**
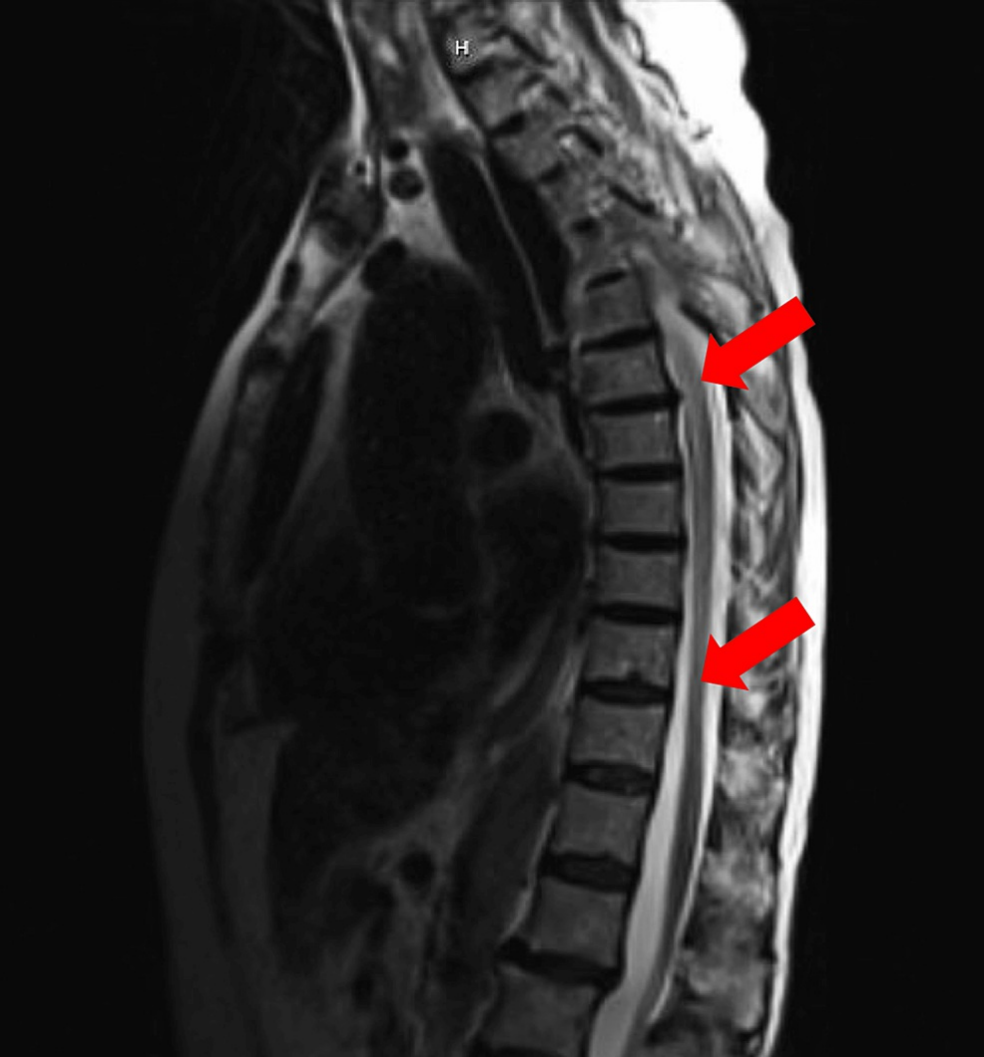
T2-weighted thoracic spine MRI, sagittal view, showing diffuse contrast enhancement (arrows) MRI: magnetic resonance imaging

Brain magnetic resonance imaging (MRI) showed scattered T2/fluid-attenuated inversion recovery (FLAIR) hyperintensities in the subcortical and periventricular white matter, interpreted to be chronic ischemic white matter changes. There was no mass effect or midline shift. T2-weighted sequence MRI of the spine was remarkable for an abnormal diffusely increased T2 signal with contrast enhancement of the lower cervical spine and most of the thoracic spine involving greater than two-thirds of the cross-sectional area (Figure 1). In the lumbar spine, there was mild grade 1 retrolisthesis of L1 in relation to L2 and disc bulge at the L4-L5 level encroaching upon the descending bilateral L5 nerve roots (Figure 2).

Lumbar puncture was also performed, and cerebrospinal fluid (CSF) analysis showed glucose of 62 mg/dL (reference range: 40-70 mg/dL), total protein of 108 mg/dL (reference range: 15-45 mg/dL), and WBC of 218 uL (reference range: 0-10 uL) with 9% polymorphonuclear WBCs. CSF serology was positive for myelin basic protein at 108.3 ng/mL (reference range: 0-5.5 ng/mL) and elevated IgG at 12.9 mg/dL (reference range: 0-6 mg/dL); however, there were no oligoclonal bands present. West Nile virus (WNV) IgG antibody was 1.63 IV (reference range: <1.29 IV), but WNV IgM antibodies were negative. NMO IgG antibody was negative in the CSF (Table 1). CSF cultures remained negative for 72 hours, and no abnormal cytology was seen on the pathology report.

**Table 1:** CSF serology panel CSF: cerebrospinal fluid

CSF serology panel	Value (reference range)
Myelin basic protein	108.3 ng/mL (0-5.5 ng/mL)
Oligoclonal bands	Negative
Immunoglobulin G	12.9 mg/dL (0-6 mg/dL)
Neuromyelitis optica IgG antibody	<1:11 (<1:11)
West Nile virus IgG antibody	1.63 (<1.29)
West Nile virus IgM antibody	Negative

The serum immunology panel showed a negative antinuclear antibody (ANA) screen. Paraneoplastic fluorescent antibody titer was indeterminate. Neuromyelitis optica IgG antibody was elevated at 24.6 U/mL (reference range: <2.9 U/mL), but there were no aquaporin 4 (AQP4) or myelin oligodendrocyte glycoprotein (MOG) antibodies (Table 2).

**Table 2:** Serum immunology panel ANA: antinuclear antibody; MOG: myelin oligodendrocyte glycoprotein; IgG: immunoglobulin G

Serum immunology panel	Value (reference range)
ANA screen	Negative
Paraneoplastic fluorescent antibody titer	Indeterminate
Neuromyelitis optica IgG antibody	24.6 U/mL (<2.9 U/mL)
MOG antibody	<1:10 (<1:10)
IgG index	0.59 (0.28-0.66)

The serum serology panel showed negative Lyme disease antibodies, and HIV was nonreactive. Notably, Coxsackie virus type B4 antibody was elevated at 1:80 (reference range: <1:10), and Echovirus type 6 antibody was elevated at >1:640 (reference range: <1:10) (Table 3).

**Table 3:** Serum serology panel

Serum serology panel	Value (reference range)
Lyme disease IgM and IgG antibody	Negative
HIV 1 and 2 antigen/antibody	Nonreactive
Coxsackie virus type B4 antibody	1:80 (<1:10)
Echovirus type 6 antibody	>1:640 (<1:10)

Based on her spine MRI findings, she was diagnosed with acute transverse myelitis (ATM). Due to the contiguous involvement of greater than three vertebrae, she was further categorized as having longitudinal extensive transverse myelitis (LETM). She was treated with intravenous methylprednisolone 1,000 mg daily for five days, alternating with plasma exchange (PLEX) therapy every other day. Normally, one of these treatments alone would have sufficed; however, they were started simultaneously due to the onset of urinary retention. She had frequent neurology checks every four hours and bladder scans every six hours. A Foley catheter was placed due to urinary retention of >800 mL, and she worked with physical therapy daily. She experienced an improvement in her weakness of the right lower extremity and was able to have the same strength in both legs by the end of treatment. Her left upper and lower limb sensory changes also improved and were back to baseline by the end of her treatment.

**Figure 3:**
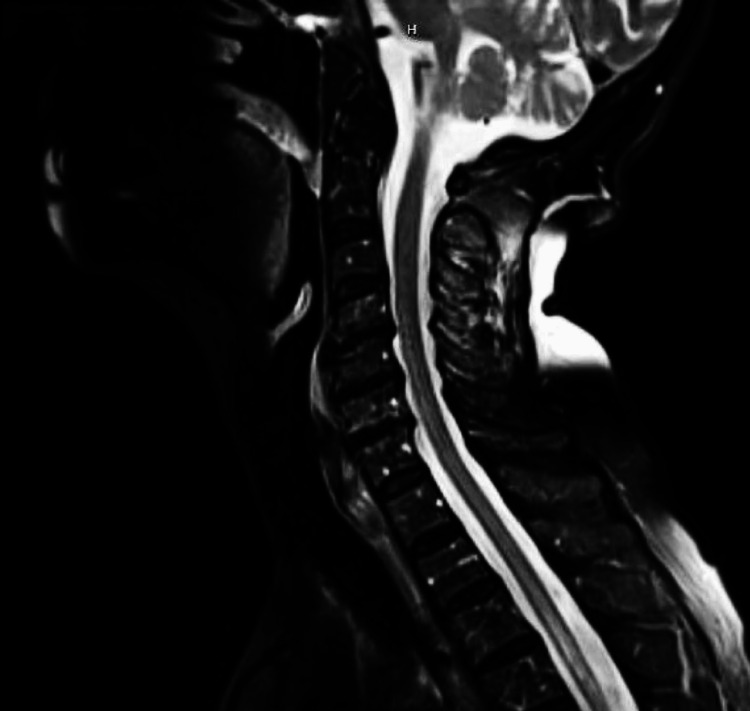
Repeat T2-weighted cervical spine MRI on day 8, sagittal view, showing nearly complete resolution of increased T2 signal at the C7 level MRI: magnetic resonance imaging

**Figure 4:**
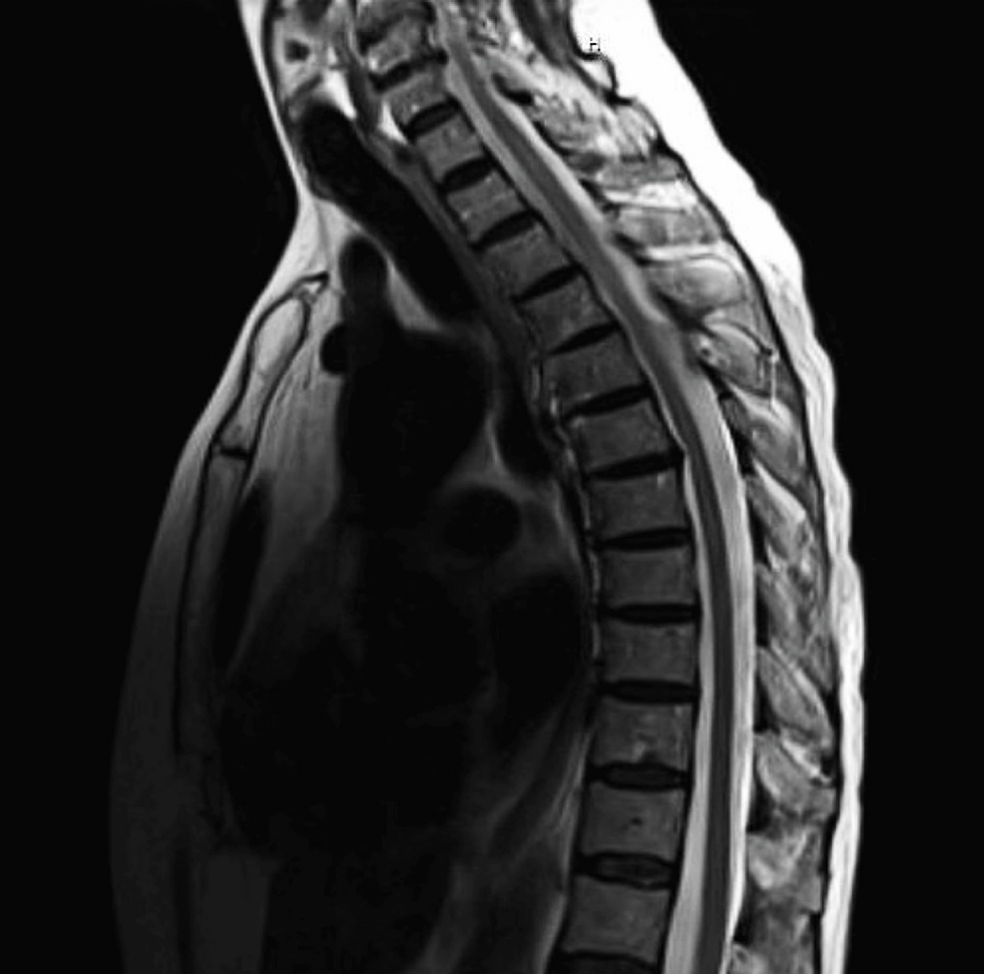
Repeat T2-weighted thoracic spine MRI on day 8, sagittal view, showing decreased contrast enhancement throughout the thoracic spine MRI: magnetic resonance imaging

She had a repeat spine MRI eight days after the initial imaging, which showed nearly complete resolution of the abnormal T2 cord signal at the C7 level and significantly improved signal in the thoracic spine that decreased from eight days prior (Figures 3, 4). Upon discharge, she was sent to an acute rehabilitation facility and recommended to follow up with neurology outpatient.

## Discussion

The diagnosis of ATM is aimed at ruling out compressive etiology with MRI and looking for signs of inflammation via the enhancement of gadolinium contrast [2]. When confined to the spinal cord without extension to the CNS or optic nerve, the diagnosis of idiopathic transverse myelitis is reached [1]. This patient had a normal brain MRI that did not show white matter changes expected to be seen in patients with MS. In addition to these diagnostic criteria, the Transverse Myelitis Consortium Working Group (TMCWG) proposed exclusive criteria that would rule out acute transverse myelitis that includes a history of previous radiation to the spine within 10 years, CNS involvement of syphilis, Lyme disease or other viral infections, thrombosis of the anterior spinal artery, brain MRI abnormalities consistent with multiple sclerosis, or clinically apparent optic neuritis [2].

This patient had interesting laboratory findings of elevated levels of NMO IgG antibody (also called aquaporin 4 antibody) in the serum, which is usually suggestive of the diagnosis of neuromyelitis optica. However, this diagnosis can only be confirmed when combined with clinical features of optic neuritis, which this patient did not have, and furthermore, her CSF levels of NMO IgG were negative. She also had elevated IgG and myelin basic protein in the CSF, which are usually associated with multiple sclerosis. However, there were no oligoclonal bands present. The patient in this case had fatigue, muscle weakness, sensory changes, and autonomic dysfunction, which could be seen with MS or NMO, but she did not have any optic or brain involvement. There have been studies showing that elevated levels of CSF MBP can be found in other neurological diseases (such as transverse myelitis, neuro-Behcet’s disease, HIV encephalopathy, or central pontine myelinolysis) due to acute myelin breakdown and may be a sign of CNS demyelination [3]. There have also been studies showing that higher levels of MBP in the CSF correlate to more active demyelination, as the serum levels tend to rapidly decrease with time [3]. She was probably in active demyelination at the time of admission, which is why her MBP was elevated.

The etiology of this patient’s diagnosis is complicated. Although her serology suggested viral infection, it could also be an incidental finding. With her history of shingles, herpes virus could have been a more likely etiology; however, this was not found in her serology. She had no systemic signs of viral illness prior to the onset of her neurological symptoms. NMO is considered an autoimmune disease, so having positive NMO IgG antibodies with her history of Hashimoto’s thyroiditis questions whether her ATM may have been from an autoimmune process. Furthermore, having an underlying autoimmune disease could have been a predisposing factor to either postinfectious or post-vaccine autoimmune phenomenon. The exact mechanism behind this type of abnormal immune response is unknown; however, when the body attacks its own cells, there will be a less effective response to infections and, vice versa, more sensitivity to potential vaccine adverse effects.

Since a host’s response to a vaccine is originally made to protect immunity, vaccines can work in similar ways to how infectious agents induce autoimmunity. Although the exact mechanism by which the postinfectious or post-vaccine autoimmunity phenomenon occurs is unknown, there are many proposed pathways. One of the most common mechanisms is molecular mimicry where there is a similar structure between infectious antigens and self-antigens [4]. Another mechanism is called epitope spreading, whereby invading antigens accelerate an ongoing autoimmune process by locally activating antigen-presenting cells (APCs) and increasing the production of antigens [5]. Additionally, infectious agents may induce autoimmune reactions through bystander activation and the expansion of autoreactive CD8 T-cells, which may be targeted to a particular virus or vaccine [6].

Between 1970 and 2009, there were 37 reported cases of vaccine-associated transverse myelitis [7]. The temporal association between vaccines and the onset of symptoms can vary from one to two weeks to as late as two months [7]. The Vaccine Adverse Event Reporting System (VAERS) Study is another comprehensive review that looked at vaccine-related transverse myelitis cases between 1985 and 2017, during which 119 cases were reported [8]. In this study, 40% of the cases had onset of symptoms within eight weeks of vaccination and 60% more than eight weeks after vaccination [8]. More recently with the onset of the COIVD-19 pandemic, there was another study that showed 43 cases of COVID-19 infection-associated ATM and three COVID-19 vaccine-induced ATM cases reported in 2021 [9]. Some of the most common vaccines associated with ATM are influenza, hepatitis B, measles, mumps, and rubella (MMR), TDaP, and human papillomavirus (HPV) [8,10].

The long-term implications of transverse myelitis may include permanent neurological damage in some patients, as much as 38.7%, as seen in the Vaccine Adverse Event Reporting System (VAERS) Study [8]. Some of these complications include constipation, urinary retention or incontinence, respiratory muscle weakness, neuropathic pain, and contractures [11]. Although most patients have resolved symptoms within three months, there is no real cure, and it can take years for some patients to get better [11]. Acute treatment consists of corticosteroid or plasma exchange therapy. Prolonged rehabilitation via physical therapy and pain management are often the mainstays of long-term treatment in these patients. Some patients with underlying autoimmune diseases may also benefit from the addition of immunosuppressive therapy, but evidence for this is limited [11].

This has led to the bigger question about the recurrence risk and the safety of using vaccines in these patients. There are many different factors that have been studied to predict the likelihood of recurrence, including brain MRI negative for findings of MS, absence of AQP4 or MOG antibodies, and having multiple distinct lesions or fusiform lesions on spine MRI extending over three or more spinal cord segments, which is also defined as longitudinal extensive transverse myelitis (LETM), like this patient [12,13]. In patients with LETM, having positive NMO IgG antibodies is also associated with a higher recurrence risk [2]. Thus, independent of vaccine adverse events, patients with such risk factors are likely to have relapse events in up to 25% of cases [2]. There is also evidence that many patients with ATM may eventually have progression to multiple sclerosis or other disorders in the neuromyelitis optica spectrum [1,2].

## Conclusions

This case of acute transverse myelitis shows a patient with several risk factors including autoimmune disease (Hashimoto’s thyroiditis), recent vaccinations, and possible viral infection exposure. Although ATM can sometimes be involved in other neurological disorders including multiple sclerosis and neuromyelitis optica, following diagnostic criteria and taking the clinical presentation into account can help with accurate diagnosis and management of this disease. In cases where possible vaccine adverse effects are suspected, it is important for these patients to be closely monitored during the course of their disease and monitor for potential recurrence. Further research into treatments may be helpful in decreasing long-term complications of ATM and preventing future relapses.

## References

[1] Sellner J, Lüthi N, Schüpbach WM (2009). Diagnostic workup of patients with acute transverse myelitis: spectrum of clinical presentation, neuroimaging and laboratory findings. Spinal Cord.

[2] Borchers AT, Gershwin ME (2012). Transverse myelitis. Autoimmun Rev.

[3] Ohta M, Ohta K, Nishimura M, Saida T (2002). Detection of myelin basic protein in cerebrospinal fluid and serum from patients with HTLV-1-associated myelopathy/tropical spastic paraparesis. Ann Clin Biochem.

[4] Blank M, Barzilai O, Shoenfeld Y (2007). Molecular mimicry and auto-immunity. Clin Rev Allergy Immunol.

[5] Lehmann PV, Forsthuber T, Miller A, Sercarz EE (1992). Spreading of T-cell autoimmunity to cryptic determinants of an autoantigen. Nature.

[6] Murali-Krishna K, Altman JD, Suresh M (1998). Counting antigen-specific CD8 T cells: a reevaluation of bystander activation during viral infection. Immunity.

[7] Agmon-Levin N, Kivity S, Szyper-Kravitz M, Shoenfeld Y (2009). Transverse myelitis and vaccines: a multi-analysis. Lupus.

[8] Shah S, Patel J, Alchaki AR, Eddin MF, Souayah N (2018). Development of transverse myelitis after vaccination, a CDC/FDA Vaccine Adverse Event Reporting System (VAERS) study, 1985-2017. Neurology.

[9] Román GC, Gracia F, Torres A, Palacios A, Gracia K, Harris D (2021). Acute transverse myelitis (ATM):clinical review of 43 patients with COVID-19-associated ATM and 3 post-vaccination ATM serious adverse events with the ChAdOx1 nCoV-19 vaccine (AZD1222). Front Immunol.

[10] Guimarães LE, Baker B, Perricone C, Shoenfeld Y (2015). Vaccines, adjuvants and autoimmunity. Pharmacol Res.

[11] Lim PA (2019). Transverse Myelitis. Essentials of physical medicine and rehabilitation.

[12] Trebst C, Raab P, Voss EV, Rommer P, Abu-Mugheisib M, Zettl UK, Stangel M (2011). Longitudinal extensive transverse myelitis--it's not all neuromyelitis optica. Nat Rev Neurol.

[13] Maillart E, Durand-Dubief F, Louapre C (2020). Outcome and risk of recurrence in a large cohort of idiopathic longitudinally extensive transverse myelitis without AQP4/MOG antibodies. J Neuroinflammation.

